# The ethical use of therapeutic touch in psychedelic-assisted therapy: a qualitative study of researcher perspectives and experiences

**DOI:** 10.1177/20451253251377191

**Published:** 2025-11-14

**Authors:** Diana McHerron, Michaela Barber, Rachel Ham, Paul Liknaitzky, Adrian Carter, John Gardner

**Affiliations:** School of Psychology, Deakin University, 221 Burwood Highway, Burwood, Melbourne, VIC 3125, Australia; Department of Psychiatry, School of Clinical Sciences, Monash University, Clayton, VIC, Australia; Department of Psychiatry, School of Clinical Sciences, Monash University, Clayton, VIC, Australia; School of Psychological Sciences, Monash University, Clayton, VIC, Australia; Department of Psychiatry, School of Clinical Sciences, Monash University, Clayton, VIC, Australia; School of Psychological Sciences, Monash University, Clayton, VIC, Australia; Department of Psychiatry, School of Clinical Sciences, Monash University, Clayton, VIC, Australia; Monash Bioethics Centre and School of Philosophical, Historical and Indigenous Studies, Monash University, Clayton, VIC, Australia; Monash Bioethics Centre and School of Philosophical, Historical and Indigenous Studies, Monash University, Clayton, VIC, Australia; School of Social Sciences, Monash University, Clayton, VIC, Australia

**Keywords:** ethics, informed consent, MDMA, psilocybin, psychedelic-assisted therapy, psychedelics, therapeutic touch

## Abstract

**Background:**

Physical touch is often included as a supportive or therapeutic tool in psychedelic-assisted therapy (PAT), involving instrumental forms of physical contact, supportive touch and somatic techniques. However, participants under the influence of psychedelics have reduced capacity to provide consent, are more suggestible and may experience and interpret therapeutic touch in ways they did not expect prior to taking the drug. Yet little research has been conducted on the considerations and approaches to therapeutic touch in clinical trials of PAT.

**Objectives:**

This study explored the experiences and perspectives of PAT researchers on the use and consent to therapeutic touch in clinical trials of PAT.

**Design:**

A qualitative study using semi-structured interviews.

**Methods:**

Sixteen PAT researchers involved in clinical trials of PAT were interviewed. Reflexive thematic analysis was used to analyse the data. The reporting of this study conforms to the Consolidated Criteria for Reporting Qualitative Research Checklist (COREQ).

**Results:**

Three themes were uncovered through reflexive thematic analysis: (1) flexible frameworks, (2) therapeutic alliance and (3) boundary management. Researchers discussed consent challenges across the broad spectrum of physical contact existing in PAT protocols at the time. Researchers indicated that consent to therapeutic touch should be established prior to the dosing sessions and continually managed throughout the course of treatment. Flexibility in consent protocols enabled researchers to interpret and approach consent through the development of a strong therapeutic alliance; however, flexibility could also lead to challenges in boundary management. Researchers emphasised the need for greater ethical guidance in instances where trial participants change their established preferences during dosing sessions, and limits on expanding consent after drug administration.

**Conclusion:**

Clear guidelines for obtaining consent, managing changing preferences and training on the management of boundary transgressions were viewed as essential for ethical research and practice of PAT.

## Plain language summary

The ethical use of therapeutic touch in psychedelic-assisted therapy

There is a growing body of research studying how mind-altering drugs, when used with therapy, may help treat mental health conditions. This type of therapy is called psychedelic-assisted therapy (PAT). During “dosing” sessions, participants take a carefully controlled psychedelic drug while receiving support from therapists. Therapists sometimes use touch, such as holding hands or reassuring support, to provide therapeutic care. Participants must consent to specific forms of touch prior to treatment.

Why This Research Matters

Getting consent for touch in PAT requires special attention because:

- Psychedelic drugs change how people think and understand information

- People might experience touch differently than expected while under drug effects

- Participants are more vulnerable and easily influenced

There are no clear guidelines for consenting participants to touch during these studies. We interviewed 16 researchers involved with PAT trials to learn about their views on obtaining and managing consent for therapeutic touch throughout treatment.

Key Findings

- Building trust between therapists and participants is very important for consent

- Agreeing to touch is not a one-time event. It requires ongoing discussion

- Therapists need better guidance about what to do when drug-affected participants change their minds about touch

Our research shows that clear guidelines are essential.

Research teams could benefit from incorporating our recommendations for:

- Managing situations when participants change their minds about touch

- Safe, ongoing communication about touch throughout treatment

- Managing clear boundaries between therapists and participants

Implications

As clinical trials of PAT expand, our findings provide a framework to inform the development of guidelines to ensure the highest level of participant safety, ethical care, and clear professional standards.

## Introduction

There has been a resurgence of clinical research investigating psychedelic-assisted therapy (PAT) in the treatment of mental illness.^1^ Psychedelic-assisted therapy typically includes three phases: preparation of patients for the therapeutic process (psychoeducation and rapport-building), one or more dosing sessions that involve the administration of a psychedelic compound under therapist supervision, and the integration of the insights and experiences gained through the dosing session. Psychedelic drugs induce significantly altered states of consciousness that are reported to elicit clinically meaningful experiences.^2–4^ Psilocybin and 3,4-methylenedioxymethamphetamine (MDMA) are the most common substances used in contemporary clinical trials. In most contemporary clinical trials since the re-emergence of this research in 1999, therapy has been delivered by a male-female therapist dyad^5^ who adopt a non-directive psychotherapeutic approach that can often include the provision of physical contact during dosing sessions.^3^

‘Therapeutic’ or ‘nurturing’^6^ touch is defined as non-sexual touch intended to provide supportive or therapeutic benefit.^7^ These forms of touch have been used within psychedelic therapy for decades.^8–10^ According to the Multidisciplinary Association for Psychedelic Studies (MAPS), a prominent group involved in developing therapy guidelines and conducting clinical trials, the use of touch, including handholding, shoulder touching, or hugging, ‘can be an important catalyst to healing’.^11,12^ PAT trial protocols define physical contact in PAT using varied terminology: ‘supportive touch’ and ‘nurturing touch’ describe similar types of touch, such as holding a participant’s hand.^11,13^ ‘Focused bodywork’ involves offering resistance for the participant to push against.^11^ The term ‘therapeutic touch’ broadly refers to supportive types of touch (e.g. holding a hand, foot or shoulder)^13^ or focused bodywork.^11^ For clarity, we categorise physical contact in PAT into three categories (Table 1): instrumental, supportive and somatic techniques. Here, we use the term ‘therapeutic touch’ in reference to all three categories, recognising that all forms of physical contact, including instrumental touch, may have psychological impacts that are experienced as helpful or harmful during altered states.

**Table 1.** Categorisation of touch in PAT.

Categories of touch	Definition	Examples
1. Instrumental	Physical contact aimed at preserving the well-being of participants and others, including conducting routine clinical or research procedures and ensuring participant safety.	Guiding a participant to the bathroom. Preventing a fall. Securing a heart-rate monitor.
2. Supportive	Physical contact intended to communicate empathic caring and contribute to the participant’s psychological well-being.	Placing a hand on a participant’s shoulder during a challenging emotional state. Holding a participant’s hand briefly during distress. Providing a hand to hold when requested by a participant.
3. Somatic techniques	Structured physical techniques performed by clinicians with appropriate credentials and specifically intended to address physical or psychological symptoms.	Applying pressure on the body (e.g., focused body work) with an object in between or providing a person with an object to apply pressure on their own body. Other formalised somatic psychotherapies.

PAT, psychedelic-assisted therapy.

In dosing sessions of PAT, participants may have periods of difficulty processing and responding to verbal support offered by the therapist. For example, during psilocybin-assisted therapy dosing sessions, participants sometimes wear eyeshades and headphones to facilitate an inward focus on thoughts, sensations and memories.^13^ As such, therapeutic touch provides an option for non-verbal communication of support,^14^ and has been reported to help patients reconnect with an ‘external reality’ in the context of challenging psychological experiences during dosing sessions.^15,16^ Furthermore, the MAPS MDMA-assisted therapy guidelines for the treatment of posttraumatic stress disorder (PTSD) caution that withholding nurturing touch when otherwise indicated during altered states could be perceived by participants as a form of ‘abuse by neglect’.^11^ Nurturing touch is also reported to provide a ‘corrective experience’ for participants during altered states who may reconnect to events from their past where they may have needed touch, but did not receive it.^11^ Further studies are needed to verify the benefits of the therapeutic touch.

The ingestion of a psychedelic drug that induces significantly altered states of consciousness raises novel ethical challenges in the provision of, and consent to, physical touch during therapy. Patients undergoing PAT may be particularly vulnerable to harm during a trial: during the acute psychedelic experience, participants may have reduced capacity for decision-making and may find it difficult to communicate their preferences and assert boundaries.^17,18^ Psychedelics can also increase suggestibility, which may persist after participants return to ordinary awareness.^19^ Many participants in trials of PAT experience significant mental distress and functional impairment, as eligibility for participation in clinical trials often necessitates a moderate to severe level of illness, for which first-line treatments have not worked.^20,21^ As such, the risks of ethical misconduct during PAT are argued to be higher than in conventional psychotherapy.^22^ Moreover, therapist behaviours that may be considered benign in conventional psychotherapy may have unpredictable impacts during PAT.^23^

Whilst touch of a hostile or sexual nature between practitioners and clients is unambiguously unethical, the ethics of other forms of touch are less clearly defined in conventional psychology and psychiatry.^24^ For example, a handshake or brief touch of the arm may generally be accepted, but anything more prolonged or intimate might be considered problematic.^25^ Ethical codes, including those of the American Psychiatric Association, the American Psychological Association, the European Psychiatric Association and the Royal Australian New Zealand College of Psychiatrists, do not make any provisions specific to touch other than prohibiting sexual contact and non-sexual contact that could be considered exploitative or demeaning.^26–29^ While the Australian Psychological Society (APS) provides ethical guidelines relating to procedures or assessments involving psychologist-client physical contact, it is unclear for which occasions physical contact is acceptable.^25^ As such, there is a lack of clinical and professional ethics guidance for conceptualising what ethical physical contact is and how to obtain informed consent to it in any setting, let alone in conjunction with the use of psychedelics.

The normalisation of physical contact between therapist and participant in PAT protocols may increase participants’ vulnerability to exploitation. Boundary transgressions in therapy range from minor deviations from established therapeutic norms that may or may not harm the therapeutic relationship to serious violations that cause harm to the client or the therapeutic process.^30^ Notably, ethical misconduct has occurred through a clinical trial of MDMA-assisted therapy employing a dyad protocol.^31^ Even in the absence of any boundary transgression or misconduct of any sort, under the influence of psychedelics, touch may be interpreted in highly variable ways and has the potential to impede therapeutic progress or cause psychological harm.^22,32–34^ The psychedelic experience not only increases participants’ vulnerability to intentional abuse but also increases the potential for unintentional or unexpected harms from altered affective states.

PAT trial participants may present with trauma histories or symptomatology, creating unique considerations for physical contact during treatment. Trauma theory demonstrates that traumatic memories encode as somatic memories in the body and implicit memory systems.^35,36^ Participants with histories of interpersonal trauma may experience selective aversion to touch^37^ and dissociation, a protective disconnection from bodily sensations.^38^ Touch may trigger the re-experiencing of traumatic memories or traumatic re-enactment, where previous traumatic patterns unconsciously reproduce.^38,39^ During PAT dosing sessions, when psychological defences may be lowered, practitioner-participant touch interactions may carry a heightened risk of activating trauma responses.^39^ These complex dynamics necessitate a trauma-informed approach to touch in PAT (e.g. via enacting principles such as safety, empowerment, choice^40^) as touch during altered states can potentially facilitate healing or inadvertently replicate past violations.^37^

Thorough and collaborative informed consent is one of the primary ways to reduce harm associated with unexpected reactions to touch during dosing. As such, the provision of informed consent is fundamental to the ethical use of therapeutic touch during PAT.^41–43^ To consent to therapeutic touch, patients must be able to comprehend information provided to them and weigh the risks and benefits.^44^ The nature of PAT complicates both aspects of consent. Firstly, the unique, context-sensitive state of consciousness produced by psychedelics makes it difficult for participants to predict what their preferences for touch will be whilst in that state, particularly if they lack previous psychedelic experience.^45,46^ Secondly, psychedelics induce cognitive, memory and executive impairments that complicate decision-making capacity during dosing sessions.^47,48^ To address these challenges, Smith and Sisti^45^ have called for an ‘enhanced’ approach to informed consent for PAT that features extended discussions with participants prior to establishing informed consent. However, in the absence of widely accepted practice standards for PAT, it is unclear how informed consent is currently obtained in trials, or what researchers consider to be important in this process, including provisions for withdrawing consent.

Existing PAT manuals^11,13,49^ state that therapists should work with participants to establish the types of touch they are comfortable with and the circumstances in which touch might be received or initiated. Touch is always optional, although some protocols suggest that therapists should encourage participants to be open to the use of touch during altered states of consciousness.^50^ While participants establish their preferences for touch during the consent process and in preparation sessions, these preferences may change during dosing. For example, participants may relive past experiences of bodily transgressions (e.g. sexual abuse) when being touched, or alternatively experience an unanticipated need to be held.^11,42^ The risk of iatrogenic harm depends upon how therapeutic touch is enacted and subjectively experienced during altered states,^18^ and how participants later view the decisions made by the therapists to uphold the boundaries that were set during preparation.

Despite these risks, there is no consensus on the conditions for consent and ethical conduct of therapeutic touch within PAT.^45,46,51,52^ Current protocols do not specify how therapists should navigate this ethical challenge when participant decision-making capacity is impaired, beyond suggesting that participant boundaries are ‘kept in mind’^13^ when therapists respond to requests for physical touch. However, researchers are navigating these questions and putting protocols into practice in clinical trials of PAT globally. There is, therefore, an urgent need to understand current practices for therapeutic touch within clinical research. This study explored researchers’ perspectives and experiences of obtaining consent to therapeutic touch in clinical trials for PAT. Three research questions guided this study:

1. What procedures for obtaining informed consent to therapeutic touch are being used in clinical trials of PAT?
2. How do these procedures engage with the unique experience and vulnerabilities associated with PAT?
3. What are researchers’ views on the challenges for obtaining informed consent to therapeutic touch in PAT?

## Methods

A qualitative interview design was chosen to capture how researchers conceptualised, implemented and reflected on their consent procedures to clinical trials of PAT. A researcher-focused approach can identify current practices regarding the provision of therapeutic touch and the management of ethical dilemmas. A critical realist standpoint was adopted for this study, acknowledging that reality exists independently from our observation of it, but that cultural, political and social factors influence representations of this reality.^53^ Researchers’ accounts of informed consent were understood to be individual interpretations of events relating to informed consent procedures, and not objective accounts of reality.

The initial study phase, including recruitment and interviewing, was mostly conducted by the first author DM, a female psychology student with training in qualitative research methods, completing her honours research project. Co-authors MB and RH were doctoral students and provisionally registered psychologists. PL leads a clinical psychedelic research group, of which RH is a member. JG is a bioethicist with a medical sociology background, and AC is a neuroethicist with a neuroscience background – both have professional relationships with individuals who research psychedelic therapies. The reporting of this study conforms to the Consolidated Criteria for Reporting Qualitative Research checklist (COREQ).^54^

### Participants

A purposive sampling approach was used to generate a diverse and rich dataset covering expertise with a variety of psychedelic substances, dosages and indications. Eligible candidates for participation were researchers currently or previously involved in current psychedelic clinical trials examining MDMA, psilocybin and lysergic acid diethylamide (LSD). This included postgraduate researchers, senior researchers holding a doctorate degree, academic and professional psychologists, or psychiatrist position. Interviewees were identified either through clinical trial databases (clinicaltrials.gov and anzctr.org.au) or through snowball sampling. Twenty-one researchers were invited to participate in the study via email. Five did not respond to the invitation. To protect anonymity, participant details such as area of research, details of the trials and specific organisational or institutional affiliations have been withheld. The final sample included 16 clinical or scientific researchers, all of whom were based at academic institutions (see Table 2). The sample size was determined based on the ‘information power’^55^ needed to satisfactorily address the research questions. Due to the purposive sampling strategy, the narrow focus on a specific population of interest and the quality and richness of the interview dialogue, particularly concerning researchers’ experiences and views on informed consent procedures within the emerging field of psychedelic science, the dataset was deemed to hold sufficient information power to facilitate a rich thematic analysis.

**Table 2.** Sample characteristics.

Variable	*n* (Total = 16)	% of Sample	Types of touch
Nationality			
Australia New Zealand United States	9 3 4	56.3 18.8 25	
Clinical researcher			
Clinical psychologist Physician (non-psychiatrist) Psychiatrist Clinical pharmacologist	4 1 1 4		
Basic Scientist			
Neuroscience Psychopharmacology Neuropharmacology Cognitive science	7 1 2 2		
Distinct clinical trials	12		
Trials with touch^a^ (clinical sample) Trials with touch (healthy participant sample) Trials without touch (clinical sample) Trials without touch (healthy participant sample)	7 2 0 3		Supportive (*n* = 7), Somatic^b^ (*n* = 4) Supportive (*n* = 2)
Study drug			
Psilocybin MDMA LSD	7 4 1		
Dosage^c^			
Micro Moderate/High	3 9	25 75	

a All clinical trials included instrumental touch.

b Somatic in reference to somatic techniques.

c Dosage was determined based on reviews of randomized controlled trials of MDMA for post-traumatic stress disorder^56^ (Low dosage = 30–49 mg, Moderate to high dosage = 50–125 mg) and psilocybin for depression^57^ (Low dosage = 0.1–1 mg; Moderate to high dosage = 10–30 mg).

LSD, lysergic acid diethylamide; MDMA, 3,4-methylenedioxymethamphetamine.

### Data collection and analysis

First author DM conducted 16 semi-structured interviews with researchers to explore current consent practices for therapeutic touch within researchers’ respective trials. Interviews followed an interview guide informed by a review of the literature and co-investigator consultation. The interview guide was pilot-tested with one researcher at the institution to evaluate the questions and identify potential misunderstandings.^58^ Initial broad, open-ended questions and prompts were provided based on concepts discussed in existing literature on therapeutic touch,^45^ including challenges to obtaining informed consent, experiences researchers have had with trial participants’ changing the parameters of their consent and perspectives towards best practice for informed consent in PAT. Interviewees discussed touch using the broad definition (‘therapeutic touch’) prevalent in the field at the time; touch intended to provide supportive or therapeutic benefit, incorporating instrumental, supportive and somatic categories of touch (see Table 1).

Interviews were 45–78 min long and conducted between July and August 2022 via Zoom from a private room within the residence of DM. Interviews were audio-recorded and transcribed by a professional agency and reviewed by DM for accuracy. Field notes were made during and after interviews. No repeat interviews were conducted. Interviewees were informed of their right to view their transcripts for accuracy and completeness. One participant requested a minor redaction from their transcript for clarity, which was implemented.

**Figure 1.**
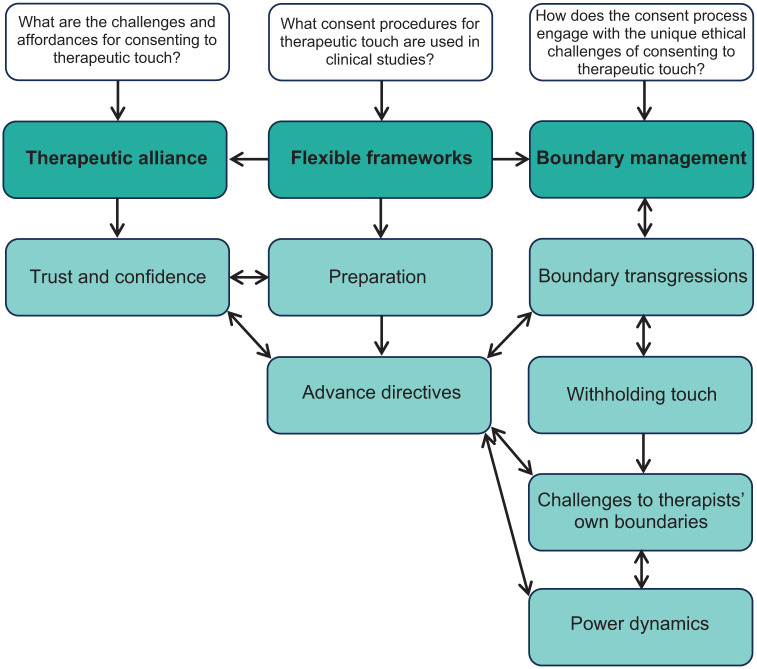
Thematic map. The research questions (white boxes), primary themes (green) and secondary themes (light green) were identified in the analysis.

Data analysis was conducted in NVivo (QSR International) and followed the six phases of reflexive thematic analysis according to Braun and Clarke.^59,60^ The primary analyst (DM) engaged deeply with the transcripts and field notes through repeated reading while maintaining a reflexivity journal to examine their held assumptions and views about touch, and how their positionality (gender, age, culture, insider status) influenced data collection and theme development.^61^ For example, author DM noted affective responses to data contributing to the theme of power dynamics, which stemmed from personal values and experiences, and discussed this with the analytical team (MB, JC, AC). Initial meaning-making involved generating potential codes that captured how interviewees constructed their experiences of consent processes in PAT.^62^ Codes were collated into 25 categories to assist with phase three, focusing on the recurring ways in which interviewees described their experiences and perspectives on consent to therapeutic touch in PAT research. The authors DM, MB, JG and AC, independently coded a sample of transcripts and then met to engage in reflexive discussions about patterns in the data. For example, clinical viewpoints initially emphasised whether trial protocols provided sufficient guidance for trial therapists to obtain and manage consent throughout PAT, while bioethical perspectives (JG, AC) highlighted potential challenges to participant autonomy during altered states. The emerging findings thus represent the team’s multidisciplinary structure, which in effect sensitised them to different dimensions of the data. Through collaborative meaning-making in coding meetings, we constructed four candidate themes: ‘flexible frameworks,’ ‘therapeutic relationship,’ ‘power dynamics,’ and ‘supervision as safeguard.’ In phase four, the data corpus was revisited, and themes were reviewed by the research team to assess whether themes provided a coherent interpretation of the data.^59^ Through reflexive engagement with the dataset, we recognised that “power dynamics” and “supervision as safeguard” were better understood as dimensions of other themes rather than standalone constructions, resulting in three main themes and seven sub themes (see Figure 1). The themes were further reviewed, defined and refined in relation to both the coded data extracts and the entire data set.^60^ The final themes were defined and labelled, and each theme was written and produced in phase six. Authors PL and RH contributed only to manuscript editing, maintaining analytical distance. De-identified illustrative quotes have been used in the presentation of results, accompanied by interviewee characteristics in the following format: (Interviewee ID, sex, profession).

## Results

Interviewees discussed touch broadly across three touch types outlined in Table 1. Three themes were derived from interviewees’ experiences and perspectives on procedures for consenting trial participants to the use of therapeutic touch in trials of PAT: (1) flexible frameworks, (2) therapeutic alliance and (3) boundary management. Each theme contains additional subthemes, as displayed in Figure 1. The themes were interrelated; for example, flexibility in the consent protocols enabled researchers to approach consent as a means of fostering the therapeutic alliance but could exacerbate challenges in boundary management.

### Flexibility within trial frameworks for consenting to therapeutic touch

This theme highlights the lack of field-wide consensus on formal processes for obtaining informed consent to therapeutic touch in PAT, which has necessitated – and afforded – the development of trial-specific approaches. One issue raised was the timing and delivery of information relevant to consent to therapeutic touch. Several interviewees expressed the view that providing participants with a written explanatory statement and consent form alone was insufficient for ethical informed consent. In this view, obtaining consent without the opportunity to discuss and possibly practice therapeutic touch was inappropriate. The participant would not have acquired sufficient knowledge to make an informed decision, including the possible forms of therapeutic touch and how it might be initiated:

Just providing the standard PICF [Participant Information and Consent Form] is probably insufficient in providing information to the participant. A more appropriate thing would be to set aside half an hour or an hour, albeit at significant extra cost, to really talk participants through what to expect. Provide them with feedback and ample opportunity to ask questions and to have them addressed before they provide their consent. Because reading a form only gets somebody so far to gaining an understanding of what they’re going to be experiencing. (Interviewee 3, M, pharmacologist)

One interviewee highlighted that their written consent forms for PAT trials included the use of therapeutic touch without specific details relating to possible forms of touch or how it could be used:

I think how things are written in our written consent form, we may use touch and participants don’t check yes or no, they just consent to the whole darn study. (Interviewee 13, F, neuroscientist)

Many interviewees expressed a preference for a staged process of consent in PAT trials, so that topics like therapeutic touch could be discussed with participants in-depth:

I think there’s definitely the possibility in my mind that we have a two-stage formal informed consent. The first in the door thing where you get the basic participant information sheet. . . and then we have a set of preparatory sessions with the participant. Then formalise some of the missing pieces of consent in an additional participant information consent form. (Interviewee 15, M, psychologist)

Interviewees emphasised the importance of preparation sessions for communicating relevant information and providing participants with an opportunity to discuss their preferences and develop ‘advance directives’ for therapeutic touch during dosing sessions. The term ‘advance directive’ was sometimes used by interviewees in relation to the process of boundary setting ahead of time. This refers to a predetermined agreement between a participant and therapist detailing the participant’s preferences for therapeutic touch during dosing sessions, when they are under the influence of an intoxicating agent and are unable to make informed decisions. To avoid conflation with legal advance directives in mental health, we have chosen to use the term ‘preference setting’. These could take the form of a blanket rule (e.g. no touch at all), while others may include contingencies, such as touch only being consensual if initiated by the participant. A few interviewees noted that within each of their distinct trials, protocol dictated that any requests for touch made by participants during dosing sessions should be declined unless covered under the preference setting, as participants lacked the necessary decision-making capacity to expand consent whilst under the acute influence of the study drug. Conversely, all interviewees agreed that participants could withdraw touch consent at any point. One interviewee described the importance of incorporating nuance and specificity into the preference setting, depending on participants’ preferences and anticipation of their mental state during dosing:

[it’s about] asking the person ahead of time what kind of touch they’d be comfortable with the therapist initiating, and what kind of touch they’d only be comfortable with if they themselves initiated it. [This] gives them scope and predictability to think, ‘I don’t think I would want a hug, but I might really badly want a hug, so I will only consent to it if I initiate it.’ I think that’s a really good way forward of separating the difference between touch that’s initiated by someone else and touch that’s initiated by the participant themselves. (Interviewee 7, F, neuropharmacologist)

Even with the added time spent on consent during the preparation sessions, the unique subjective nature of psychedelic experience was seen as a challenge to obtaining consent sought from participants during normal waking consciousness. Interviewees noted the importance of discussing with participants the possibility that their preferences might change due to the acute drug effects, to the extent that even the same type of touch demonstrated during preparation sessions might be experienced differently during dosing:

It needs to be explained in the preparatory session that you might have a very, very strong aversion to be touched in your current state, but that state may well change during the intoxication of the agent. ‘Would you ever foresee or be able to foresee a situation where you do want to have your hand held by a therapist?’. (Interviewee 14, M, psychiatrist)

Overall, obtaining informed consent to therapeutic touch was described by interviewees as a process that needed to be more involved and detailed than a written explanatory statement and a binary yes/no response. Ideally, it would involve experiential learning and preference setting through preparation sessions to form nuanced and flexible practices. However, even this more involved process of consent could be complicated by the nature of a psychedelic-induced altered state.

### Therapeutic alliance

The therapeutic relationship between the trial participant and their therapist dyad was seen as a key component of obtaining informed consent to therapeutic touch. A foundation of trust between participants and therapists was seen by some interviewees as a necessary precursor for participant disclosure of their relevant personal history, for their preferences regarding touch, and supporting their confidence to undergo the psychedelic experience. One interviewee explained that discussing participants’ history with physical touch was important to consider, due to the potential influence that past experiences may have on the ways touch is interpreted by participants before, during and after dosing:

If you could think about any of the things that touch can communicate, just with people in general, well, those things can happen in session and they can be good and they can be bad. Touch can also be an assault, it can be intrusive, it can be sexual, it can be unwelcome, it can be violent, it can be many negative things as well. It can be confusing. So, all of the functions of touch that it has socially can show up in the therapeutic relationship as well and those are the kinds of things we’re trying to consider. Interviewee 12, M, psychologist)

This quote illustrates that the therapeutic alliance was viewed as a site in which participants’ histories with touch and relational trauma might re-emerge. Building a trusting, working relationship was essential to supporting participant disclosure of their experiences, preferences and needs. Several interviewees also noted that, in addition to their personal history, participants’ identity and cultural background were important aspects to discuss during the consent process, as experiences of racial injustice, or marginalisation due to sexual/gender identification, may also influence the reception of touch.

. . .for example, people with certain identities or in certain cultural groups may have different experience with touch that may be relevant to how it is experienced. . . let’s say I’m a white male and I’m working with a Black, female client, or [. . .]someone with those sorts of backgrounds have often had experiences where white people feel entitled to touch them in various ways or may have actually been physically assaulted by white people. Or other sorts of assaults that are associated with physical control. Understanding people’s identity and their cultural background as it relates to touch is an important part of really understanding whether you truly have consent and what true consent would be in the context of a therapeutic relationship. (Interviewee 12, M, psychologist)

Interviewees illuminated participant preferences by helping them understand and articulate what therapeutic touch is and engendering a sense of safety and trust in the therapists.

### Boundary management

Interviewees described the importance of managing boundaries between therapists and participants when consenting to therapeutic touch. Boundary management was defined by participants as providing care according to established professional, personal and emotional boundaries. This was an ongoing, evolving process, wherein touch preferences were first established in preparation for the dosing session, but open to negotiation between therapist and participant over the course of the trial. Interviewees noted the responsibility of the therapist to uphold the pre-established boundaries of the participant during dosing sessions. However, the discussions highlighted four challenges in boundary management during consent and dosing process: (1) boundary transgressions, (2) withholding touch, (3) challenges to therapists’ boundaries and (4) power dynamics.

#### Boundary transgressions

Hypothetical examples of boundary transgressions in PAT were given by several interviewees, such as participants having a negative reaction to touch during an altered state, or if a therapist was unable to fulfil a participants’ request for more or different touch to what they had previously consented to. The risk of boundary transgressions in PAT was seen to be high. Even when the therapist provides care exactly as agreed upon prior to the dosing session, a participant experiencing intense and fluctuating cognitive, perceptual, and emotional changes may interpret this care as unhelpful or even deliberately harmful:

[. . .] anecdotally I am conscious of how some people who didn’t mind having their hand held very much resented when somebody tried to gently guide them to the bathroom, just by touching them on the shoulder and telling them this is the way, not that way. They had a very difficult experience after that. So, things which would appear to be trivial, become highly significant and vice versa. (Interviewee 14, M, psychiatrist)

This quote illustrates that even instrumental forms of touch that may be necessary for participant safety, or that the participant consented to before, may be experienced negatively and therefore felt as a boundary transgression. Some interviewees emphasised the need for therapists to carefully address the topic of touch with participants through using precise and thoughtful communication during dosing sessions:

[. . .] Most of the time you would be checking on consent before you would initiate touch. You might say something like, ‘how would it feel right now for me to put my hand on your shoulder?’. . . we were coached to ask questions like, ‘how would it be if I?’, rather than ask, ‘is it okay if I?’, so the person has more of a sense of being able to describe their reaction, rather than feeling pressured to say yes to a request. (Interviewee 12, M, psychologist)

This quote illustrates that some protocols acknowledge the heightened suggestibility of participants during an altered state of consciousness and consequently substitute direct requests with open-ended reflections to inform therapists’ decision-making during a session.

#### Withholding touch

Interviewees also noted the potential negative impact on participants of withholding touch, particularly in the context of participants undergoing challenging experiences. While all interviewees agreed that participant requests for expanding consent during psychedelic states should not be accommodated due to potential impairments in decision-making, one interviewee shared their perspective on the potential therapeutic ramifications of withholding participant-requested touch in session:

I have a lot of sympathy for the view that it could be unethical to withhold touch in a high psychedelic trip for some people. It could reinforce difficult lessons that people can draw from psychedelic experiences like they were overwhelmed, they were out of control, and they were abandoned. (Interviewee 15, M, psychologist)

However, some interviewees noted the possibility of non-touch alternatives to therapeutic touch that maintain the boundaries established in preparation:

[. . .] rather than you touching them, as a way for you to show caring but without touch, you could wrap them in a blanket. . . or say they have a dog, you could have them picture that the dog’s there with them and they’re petting their dog. There’s alternatives that can be used to try to respond to whatever is that desire that’s coming up, that don’t have to involve actually touching the person in that moment. (Interviewee 12, M, psychologist)

#### Challenges to therapists’ own boundaries

Interviewees noted the importance of PAT therapists knowing and maintaining their own boundaries relating to initiating and receiving touch from participants, highlighting that participant-initiated touch in dosing sessions also requires the therapist’s consent:

I think the negotiation’s two-way, and so somebody that hasn’t had MDMA before probably won’t realise that they’re going to feel like hugging everyone in the room. So, negotiating with the participant what the therapists are comfortable with as well. (Interviewee 9, M, psychologist)

Much like the ongoing, reflective consent process that many interviewees recommended for participants, it was acknowledged that to practice ethically, therapists needed to be mindful of their own histories, preferences, and competencies regarding the provision of therapeutic touch:

[. . .] the therapist needs to only give touch in a way that is comfortable for them and in line with their expertise. They need to track their motivations and they need to track their affective state for it. . . we work through ways in which we cannot fulfil a request for touch if we don’t want to, without producing a sense of rejection or abandonment in the participant. (Interviewee 15, M, psychologist)

#### Power dynamics

Interviewees reflected on their feelings and responses towards the power differential between therapists and trial participants, and how this can influence consent to therapeutic touch. Many interviewees referenced media coverage of ethical violations in MDMA-assisted psychotherapy research.^63^ Some interviewees noted that there had been discussion within their research teams about the ethical issues raised:

Yeah, it’s a really big problem. It’s being discussed at the moment. What the solution is, is not going to be perfect. However, you can probably draw upon what other fields have already been dealing with, like what the psychotherapy fields have already been dealing with. We can look back at things like hypnotherapy or those repressed memories, the history of all of those sorts of things to maybe learn lessons about where not to go and what has worked and what hasn’t. (Interviewee 6, F, neuropharmacologist)

One interviewee reported processes in their trial protocol designed to mitigate the effects of the therapist-participant power differential:

Our senior research officer will call a participant after their first dosing session, to check in. That was predominantly put in place so that there was a third party that participants could talk to, if they felt uncomfortable with things like therapeutic touch in their [dosing session] but they felt unable to talk to their therapists about that. (Interviewee 2, F, psychologist)

This quote illustrates how researchers are incorporating additional processes involving a neutral researcher outside of the therapist-dyad to promote the communication of boundary transgressions. This external researcher provides participants with an alternative channel outside of the therapist dyad to communicate and revise their touch preferences. A sense of trust in the research team was reported by some interviewees as a factor in consent, as trust in the system in which the research is being conducted may also confer trust in the therapists involved in the research. Participants may be better equipped to engage in the consenting process if they believe their therapists can provide the necessary support during distressing psychedelic experiences:

How our participant relates to that individual or to ourselves and how that gives them confidence that, I don’t have to worry if something goes wrong, this person is here for me. (Interviewee 1, neuroscientist)

Researcher involvement throughout the consent process may introduce a layer of accountability by the research team, who are often viewed as more ‘external’ to the therapeutic process. Their role as non-biased supporters may add a measure of safety and facilitate open communication, which is an important consideration for future clinical trials involving therapeutic touch.

## Discussion

Therapeutic touch is included in some protocols for PAT,^11,13,49,50^ but there is limited ethical guidance for consenting participants and patients to this aspect of treatment. This study is the first to examine researchers’ perspectives and experiences of consenting participants to therapeutic touch in clinical trials of PAT.

### The use of preference setting in consenting to the therapeutic touch

A key finding of this study is that during preparation sessions, trial participants’ preferences for touch are often written and recorded by the research team to guide decisions made by therapists during dosing sessions. Based on these findings, we strongly recommend the development and formal implementation of a standardised protocol for recording and enacting touch preferences throughout the therapy process (see Table 3 for a list of recommendations). Advance directives in psychiatric care help individuals with episodic mental illnesses, such as bipolar disorder, and set treatment preferences for episodes of reduced decision-making capacity.^64^ These preferences may be influenced by previous experiences of these episodes and the care they received.

**Table 3.** Recommendations for obtaining and managing consent to therapeutic touch.

Core domains	Recommendation
1. Preference Setting	• The purpose, risks and intended benefits of touch (instrumental, supportive and somatic techniques) should be clearly communicated to participants ahead of dosing sessions. Discuss specific trauma-informed non-touch alternatives such as psychodynamic containment, grounding exercises, guided imagery. • Establish participant preferences for touch during preparation (e.g. who touches, who initiates, how non-consent is communicated). The possibility of altered preferences during dosing should be considered (e.g. no therapeutic touch even if patient-initiated, or no therapeutic touch unless patient-initiated and if it is highly constrained). • Develop specific protocols for managing preference shifts during sessions, including predetermined non-verbal signals when communication is impaired. • Implement a standardised protocol for recording and enacting touch preferences throughout the therapy process. • Explore with participants their frame of reference for touch, including past experiences, race, culture, age, gender, sexuality, trauma history, and relationships to oppression, power and privilege.
2. Therapist Competencies	• Therapists who administer touch receive specialised training to develop skills in negotiating the consenting process and risks of boundary transgressions. Training includes specific decision-making frameworks for responding to edge cases (e.g. dissociative states, trauma reactivation) and addressing moral distress when withholding requested touch. • Ensure therapists are proficient in trauma-informed non-touch interventions that maintain therapeutic connection when physical contact is inappropriate. • Respectful engagement with client autonomy (e.g. responding to participant hesitation or disagreement with curiosity and collaboration rather than pathologising it as resistance.)
3. Boundary Management	• As a default position during dosing sessions, therapists do not provide touch beyond what was agreed to during preference setting, even if requested by the participant. Experiences of moral injury to therapists are addressed in supervision or training. • Participants can decline or retract their consent to therapeutic touch whilst under the acute effects of the study drug, and the therapist must uphold this. • Practice the communication of non-consent, and regularly check-in with participants to confirm or adjust consent (with consideration to verbal and non-verbal cues). Implement context-sensitive methods for confirming ongoing consent that account for altered communication abilities during different phases of the psychedelic experience. • Provide participants with ongoing channels outside of the therapeutic relationship/s to (1) discuss their experience of and preferences for touch and (2) address barriers to communication, such as power imbalances within the therapist-patient relationship.
4. Research and Collaboration	• Conduct research to understand participants’ and therapists’ experiences of withholding touch during dosing sessions to inform ethical decision-making. • Collaboration between clinical psychedelic groups to develop shared ethical and professional standards for therapeutic touch, informed by a diversity in consenting experience across a variety of clinical populations/drug conditions.

However, a key difference between the conventional use of preference setting (i.e. advance directives) and those described in clinical trials of PAT is that participants are unlikely to have had any experiential knowledge of psychedelic-induced states to draw on to inform their preferences regarding therapeutic touch during PAT.^65^ Interviewees spoke of attempting to overcome this knowledge gap by using preparation sessions and providing experiential examples of touch (e.g. a therapist-initiated touch on a participant’s shoulder). However, this approach does not account for the qualitative differences in touch experienced during altered states. The difficulty of imagining a psychedelic experience complicates informed consent, as the experience itself may be essential for making informed, autonomous decisions.^66,67^ It has been suggested that partial mental simulation, supplemented by detailed information about the potential for epistemic and personal transformations, may be considered sufficient for consent to PAT.^45,67^ Research indicates that PAT can be transformative in nature, enabling participants to make decisions that are aligned with their values.^67^ Thus, even though patients cannot fully anticipate the psychedelic experience, they can understand the potential transformative nature of these experiences and subsequently inform their decision to consent to PAT. At present, participants may consent to therapeutic touch that is later experienced as intrusive or distressing due to the unpredictable nature of psychedelic experiences.

The strength of the therapeutic relationship is also thought to be an important factor in supporting positive outcomes in PAT generally, by providing a safe space for the participant to open up to the psychedelic experience.^68^ Interviewees spoke of systems for participants to report their experiences of therapeutic touch with research team members outside their therapist dyad, and discuss developments or changes in their preferences over the course of the trial. Although extended interactions with therapists have the potential to assist clients in making informed decisions, they also risk subjecting clients to undue pressure, particularly when therapists’ personal views on the benefits or harms of therapeutic touch may inform their guidance. Thus, while the extended engagement with therapists may be supportive of client therapeutic outcomes and informed decision making, awareness of the potential imposition of therapists’ biases may be important to ensure participants’ autonomy and safety throughout the process.

The importance of trust and a sense of security within the therapeutic alliance brings to the fore the fallacy that the informed consent process is only a rational cost-versus-benefit calculation.^69^ Consenting to therapeutic touch involves the ongoing activation of emotional, affective sensibilities, further shaped by subjective psychedelic experiences. Relational ethics conceptualises consent as an ongoing dialogue, rather than a discrete event.^70^ This framework positions practitioner and client co-creating a therapeutic space where the relationship itself evolves throughout the encounter, requiring continuous attention to power dynamics and safety rather than relying on initial consent alone.^71^ Unlike approaches emphasising autonomy as individual and static, relational ethics highlights how autonomy is exercised within relationships of interdependence, where trust and reciprocity create conditions for meaningful consent during altered states.^70^ Care ethics similarly recognises the importance of attentiveness to evolving patient needs and responsiveness within relationships characterised by dependency.^72^ These frameworks may help explain our study findings that consent processes appear to involve ongoing negotiation and are shaped by the therapeutic relationship, suggesting the need for guidance that acknowledges these evolving and relational dimensions in the ethical provision of therapeutic touch.

### Preventing boundary violations

Interviewees held varied views on managing challenges to carrying out preference setting during dosing sessions. All suggested that during altered states, preference setting should be upheld, and any requests to expand touch consent should be denied. This approach aligns with the precautionary principle, which advocates for protective measures in the presence of potential risks despite incomplete scientific understanding, and has been recommended in such scenarios.^22,73^ However, some interviewees expressed concern that withholding touch from distressed participants who were actively requesting it could be counter-therapeutic or harmful. This perspective may reflect therapists’ moral imperative to provide care, and subsequently, their moral distress when they cannot provide the care they believe is needed.^74^ Moral distress may be experienced by clinicians when preference setting constrains the provision of in-the-moment therapeutic care, including supportive touch, which may result in feelings of guilt and powerlessness.^75,76^ To minimise this issue, therapists could establish consent for participant-initiated touch during the preference setting, allowing for its provision upon request.

Some interviewees noted that boundary challenges in PAT are bidirectional. Therapists may receive or initiate touch that creates discomfort, arising from transference, regression, or disinhibition during altered states. Therapists must simultaneously maintain boundaries while responding therapeutically to distress.^77^ This complex dynamic may expose therapists to secondary traumatic stress and countertransference reactions, particularly when navigating difficult consent decisions about touch. While protocols discuss therapist self-care to prevent vicarious traumatisation and compassion fatigue, it is unclear how therapists navigate their own boundaries when feeling uncomfortable or compromised, without disrupting therapeutic rapport or inducing participant shame.^11,13^ Our findings highlight the need for supervision addressing vicarious trauma and providing consultation for complex boundary scenarios, important for therapist wellbeing and ethical practice.

The development of consensus-driven guidelines could clarify these complex scenarios, reducing ambiguity and better preparing therapists and participants. In this case, consensus guidelines might delineate the acceptability of participant-initiated versus therapist-initiated touch, thereby alleviating therapist moral distress by bolstering confidence in decision-making. Understanding whether moral distress is a common experience for trial therapists would inform the training and supervision of psychedelic therapists to manage these conflicting demands in a client-centred and ethical manner. The findings also necessitate a critical examination of whether withholding touch is ethically unacceptable in specific contexts or adversely affects treatment outcomes. Establishing a position on withholding or withdrawing touch can provide therapists and trials with a justified basis for their clinical decision-making in specific scenarios.

Another challenge to boundary management raised by interviewees was scenarios wherein participants had become distressed in response to touch that they had previously consented to. During preparation sessions, practicing touch between therapist and patient is intended to explore expectations and boundaries for the experience of touch during altered states. However, participants’ expectations regarding touch may be different from those experienced during an altered state. Preference setting may not be sufficient to protect participants from negative touch experiences during PAT. Trial protocols include mechanisms for participants to readily communicate changing boundaries during sessions.^13^ Visual cues, hand signals, or other non-verbal communication systems could provide participants greater agency during moments when linguistic capacity is impaired. Additionally, some interviewees mentioned non-touch support strategies for when touch is contraindicated. Psychodynamic containment techniques and trauma-informed non-contact methods, such as imagery or grounding techniques, may establish psychological safety while adhering to consent parameters and addressing participant distress.^78^ Other supportive approaches could include the use of comfort items such as weighted blankets. Overall, these findings raise important questions regarding whether the risk of inadvertent boundary transgressions is inevitable, and if so, how to best mitigate resulting moral harms. It may also suggest that equal attention is paid to practicing ways of communicating both consent and non-consent to promote attunement between therapist and patient throughout dosing sessions.

While some theorists suggest that navigating these incidents carefully can strengthen the therapeutic alliance,^77,79,80^ incidents of boundary transgressions must be approached with caution, particularly given the potential for abuse in scenarios where participants are encouraged to open up to the psychedelic experience during intense and vulnerable states.^67^ Normative criteria of boundary violations in psychedelic research include obvious cases of sexual abuse in clinical trials and clear ethical breaches such as non-consensual physical contact, coercion, or exploitation.^30^ Effective boundary management may also include monitoring participant experiences of unhelpful touch during altered states. This process can disambiguate potential boundary crossings that are at heightened risk of becoming violations or otherwise impacting the therapeutic environment and participant safety. Additionally, it may be important to understand when and how touch is helpful to participants so that touch can be used judiciously by therapists. The development of agreed-upon definitions for boundary crossings versus violations is also needed to assist therapists and research teams in evaluating their decision-making against established norms.

The capacity for therapists to recognise and address boundary transgressions when they occur is complicated by the power differential between participants and therapists. To address this concern, researchers in this study described additional safeguards in their trial protocol for participants to discuss their experiences of touch with a neutral researcher outside of the therapist dyad. This was implemented in response to the media reports of ethical violations in clinical trials for PAT.^31^ This finding follows a similar approach to the ‘ethics-in-process’ model for consent used in dementia research.^81^ This model acknowledges the situated vulnerability of research participants with dementia who possess impaired decision-making capacity, limiting the dependency of participants on the researcher and the research context.^82^ The findings of this study indicate that such an ethics-in-process model could benefit the field of psychedelic research and practice.

Other potential measures to reduce harm include video recording of dosing sessions with independent review.^11^ Video recording may support procedural accountability and provide participant reassurance during consent discussions.^83^ However, recording raises privacy concerns given the personal nature of treatment sessions and the potential impact on the therapeutic setting.

### Implications for clinical trials in psychedelic-assisted therapies

Ethical guidelines and frameworks for researchers and clinicians using physical contact in PAT clinical trials are urgently needed. Clinical trial protocols should address the potential for boundary transgressions and provide training to develop trial *therapist competencies* in navigating this ever-present risk between therapist and patient. This training should include practical skills for responding to preference shifts mid-session, recognising non-verbal withdrawal of consent and employing trauma-informed non-touch alternatives when indicated. Preparation sessions involving *preference setting* should not only prepare participants for the psychedelic experience, but also for identifying and communicating their boundaries with therapists during the acute drug phase. These sessions should be structured to include explicit practice of boundary communication – both consent and non-consent – and should acknowledge how cultural background, trauma history and power dynamics influence touch experiences. Barriers to communication, such as power imbalances within the clinician-patient relationship, may be addressed through additional safeguards such as third-party support from a neutral researcher, as part of a broader strategy for *boundary management.*

To prevent the experience of moral distress for clinicians in therapeutic trials, the development of ethical guidance for therapists on how to respond to psychological distress when touch is not consented to may ameliorate this challenge.^6,41,45^ This may involve examining trial participants’ experiences of challenging altered states when touch is withheld to support the therapist’s ethical decision-making.^45^

Enhanced *research and collaboration* between clinical psychedelic research groups may support the development of ethical and professional standards for therapeutic touch informed by a diversity in consenting experience across a variety of clinical populations/drug conditions. This may support successful translation of PAT to community settings,^84^ and efforts to incorporate therapeutic touch within a psychological-medical framework. To support future clinical trials incorporating these implications, key recommendations derived from this study are summarised in Table 3.

### Limitations and future directions

This study provides world-first perspectives from key researchers in psilocybin and MDMA-assisted therapies regarding the current consent processes to therapeutic touch in different clinical populations. Future research will need to examine patients’ experiences of therapeutic touch during PAT to provide a complete picture. Also, cultural and socio-political factors can impact healthcare professionals and patients’ experience of touch in other care contexts.^85^ These findings also report the views of researchers in developed, predominantly English-speaking countries, reflecting biases within a field where staff and research participants are predominated by White, heteronormative cultures.^86^ These views may not reflect the perspectives and experiences of other identities (e.g. queer, Indigenous and people of colour) who are largely underrepresented in psychedelic research.^86,87^ Future research should examine the perspectives of people from diverse backgrounds to understand the role of culture in consenting to therapeutic touch.^52^ Our findings also suggest that future research might consider adopting the term ‘supportive touch’ in preference to ‘therapeutic touch’, as this terminology acknowledges therapeutic intent without presupposing beneficial outcomes.

## Conclusion

These study findings of clinician and non-clinician researcher perspectives suggest that future consent and ethical guidelines to therapeutic touch incorporate appropriate flexibility, such as imposing strict limits on expanding the parameters of consent whilst participants are in an altered state of consciousness, whilst maintaining their right to withdraw consent at any time. The findings also suggest incorporating external channels for participants to further refine their touch preferences outside the therapeutic relationship. The perspectives gathered highlight a need for further research investigating how both trial participants and researchers view the process of consenting to therapeutic touch. As these practices evolve, supervision and oversight will support decision-making by clinician and participants. Future research may also interrogate ideas raised in our study findings, such as whether the act of withholding touch constitutes a form of harm. Clinical trial protocols should develop clear processes that acknowledge circumstances where therapeutic touch might be indicated. These protocols should include therapist-patient education specifically on the management of boundary transgressions, and participants’ ongoing right to withdraw consent at any time. The methods of delivering therapeutic touch should also be further developed, tested, deployed and shared.

## Supplemental Material

sj-docx-1-tpp-10.1177_20451253251377191 – Supplemental material for The ethical use of therapeutic touch in psychedelic-assisted therapy: a qualitative study of researcher perspectives and experiences Supplemental material, sj-docx-1-tpp-10.1177_20451253251377191 for The ethical use of therapeutic touch in psychedelic-assisted therapy: a qualitative study of researcher perspectives and experiences by Diana McHerron, Michaela Barber, Rachel Ham, Paul Liknaitzky, Adrian Carter and John Gardner in Therapeutic Advances in Psychopharmacology

sj-pdf-2-tpp-10.1177_20451253251377191 – Supplemental material for The ethical use of therapeutic touch in psychedelic-assisted therapy: a qualitative study of researcher perspectives and experiences Supplemental material, sj-pdf-2-tpp-10.1177_20451253251377191 for The ethical use of therapeutic touch in psychedelic-assisted therapy: a qualitative study of researcher perspectives and experiences by Diana McHerron, Michaela Barber, Rachel Ham, Paul Liknaitzky, Adrian Carter and John Gardner in Therapeutic Advances in Psychopharmacology

## Declarations

**Ethics approval and consent to participate:** Ethics approval was obtained from the Monash Human Research Ethics Committee (ID: 32651). Written consent to conduct and publish the study was collected from participants before each interview.

**Consent for publication:** Not applicable.

**Availability of data and materials:** The datasets generated and analysed in this study are not publicly accessible, as the raw data cannot be anonymised, and there is a risk that the privacy of individual participants could be compromised.
