# Supplementary material for: The ethical use of therapeutic touch in psychedelic-assisted therapy: a qualitative study of researcher perspectives and experiences
Source: Ther Adv Psychopharmacol. 2025 Nov 14;15:20451253251377191. doi: 10.1177/20451253251377191 (PMC12618852; doi:10.1177/20451253251377191)
Supplement: sj-docx-1-tpp-10.1177_20451253251377191 – Supplemental material for The ethical use of therapeutic touch in psychedelic-assisted therapy: a qualitative study of researcher perspectives and experiences [file sj-docx-1-tpp-10.1177_20451253251377191.docx]

**Interview Schedule**

**Background Questions:**

● We will begin with a few questions about your professional background and expertise.

- What is your professional background or discipline?
- How long have you been involved in psychedelic research?
- What sort of psychedelic research have you been involved in? [prompts: basic scientific research, clinical trials, other]
- Which psychedelic drugs have you been studying?
- Which health conditions have you been studying?

1. **What are the key points that need to be discussed with participants at consent for psychedelic research?**
2. **What are some of the key challenges of consenting participants to clinical trials with psychedelic assisted therapy?**
3. **Do you use therapeutic touch in your research trials?**
   1. **(*if no*: Do you think therapeutic touch can be beneficial in psychedelic assisted therapy?** *If so, how?*)

- Have you experienced any challenges around touch during a psychedelic session?
- What are the challenges of therapeutic touch during consent?
  - Have you had experience of someone revoking consent to touch in session?

1. **Can you tell me about any ethical dilemmas that have come up for you in psychedelic research?**

- Why do you think they occurred?
- How did you deal with them?
- How do you navigate those ethical dilemmas in informed consent?

1. **What is important to ensuring rigorous informed consent processes in psychedelic research?** More concrete: ***What strategies or protocols do you use to ensure a rigorous/consistent consent process?***

- Do you think informed consent standards need to be improved? What are the areas of improvement?
- How do you think informed consent standards should be improved in psychedelic research?

1. **Is there anything else that you would like to add that we haven’t discussed yet?**
2. **We are looking to recruit other researchers studying psychedelics. Is there anyone that you would recommend we speak to?**
3. **Finally, I would be happy to receive any feedback on how this interview went, here or via email. Thanks for your involvement.**
